# Supplementary material for: Sub-Regional Variation and Characteristics of Cabernet Sauvignon Wines in the Eastern Foothills of the Helan Mountain: A Perspective from Phenolics, Visual Properties and Mouthfeel
Source: Foods. 2023 Mar 3;12(5):1081. doi: 10.3390/foods12051081 (PMC10000446; doi:10.3390/foods12051081)
Supplement: Supplementary file 1 [file foods-12-01081-s001.zip › SUPPLEMENTARY INFORMATION.pdf]

SUPPLEMENTARY INFORMATION

Table of Contents

Table S1A. The information and basic composition and $L*$, $a*$, and $b*$ values of all wine samples.

Table S1B. The statistical summary of basic physical and chemical indexes of Cabernet Sauvignon wines from six sub-regions of EFHM.

Table S2A. The materials and scales for training in CATA.

Table S2B. The CATA frequency of 71 Cabernet Sauvignon wines from the six sub-regions of EFHM.

Table S2C. The QDA result of 71 Cabernet Sauvignon wines from the six sub-regions of EFHM.

Table S3. The historic terroir parameter of the six sub-regions of EFHM.

Figure S1. Cross-validated score plot for the OPLS-DA model based on the concentrations of phenolic compounds in Cabernet Sauvignon wines from six sub-regions of EFHM.

Figure S2. Validation plot obtained from 200-time permutation tests for the OPLS-DA model based on the concentrations of phenolic compounds in Cabernet Sauvignon wines from six sub-regions of EFHM.

Table S4. Concentration of non-anthocyanin phenolic compound in Cabernet Sauvignon wines from six sub-regions of EFHM.

Table S5. Concentration of anthocyanin in Cabernet Sauvignon wines from six sub-regions of EFHM.

Table S6. Concentration of anthocyanin derivative in Cabernet Sauvignon wines from six sub-regions of EFHM.

Table S1A. The information and basic composition and *L**, *a**, and *b** values of all wine samples.

NO	Region	Variety	Vintage	Alcohol level (%,*v*/*v*)	Residual sugar (g/L)	pH	Total acidity (g/L)	Volatile acidity (g/L)	*L**	*a**	*b**
SZS-1	Shizuishan	85% Cabernet Sauvignon, 15% Cabernet Franc	2015	14.65±0.00	3.80±0.00	4.04±0.00	5.50±0.00	0.87±0.00	49.29±0.87	40.58±0.26	33.27±0.40
SZS-2	Shizuishan	100% Cabernet Sauvignon	2019	14.48±0.00	3.07±0.06	3.81±0.00	5.70±0.00	0.76±0.00	53.47±0.15	41.11±0.10	23.76±0.09
HL-1	Helan	80% Cabernet Sauvignon, 20% Merlot	2016	15.08±0.00	2.47±0.06	3.99±0.01	5.70±0.00	0.81±0.01	54.74±0.16	37.50±0.10	31.99±0.12
HL-2	Helan	80% Cabernet Sauvignon, 20% Merlot	2017	14.29±0.00	1.83±0.06	3.95±0.00	5.70±0.00	0.65±0.00	57.72±1.29	37.12±0.34	28.82±0.25
HL-3	Helan	100% Cabernet Sauvignon	2017	15.16±0.01	2.93±0.06	3.90±0.01	5.50±0.00	0.61±0.00	49.84±0.07	42.16±0.07	25.78±0.11
HL-4	Helan	100% Cabernet Sauvignon	2020	15.74±0.01	3.27±0.06	4.04±0.00	5.53±0.06	0.65±0.00	44.48±0.10	45.64±0.10	26.72±0.17
HL-5	Helan	100% Cabernet Sauvignon	2021	15.49±0.00	10.23±0.06	3.82±0.00	7.60±0.00	0.53±0.01	23.72±0.23	62.74±0.27	13.35±0.33
HL-6	Helan	100% Cabernet Sauvignon	2019	15.58±0.00	3.90±0.10	3.76±0.00	5.90±0.00	0.67±0.01	40.37±0.11	49.70±0.07	20.65±0.18
HL-7	Helan	100% Cabernet Sauvignon	2019	15.40±0.00	3.90±0.00	3.77±0.00	5.80±0.00	0.46±0.00	39.12±0.07	52.50±0.04	21.72±0.08
HL-8	Helan	100% Cabernet Sauvignon	2018	15.53±0.01	5.87±0.06	3.79±0.00	6.00±0.00	0.55±0.01	52.59±0.23	42.57±0.14	23.20±0.07
HL-9	Helan	100% Cabernet Sauvignon	2019	15.34±0.01	3.80±0.00	3.81±0.01	6.20±0.00	0.70±0.01	33.41±0.35	55.27±0.30	21.06±0.21
HL-10	Helan	100% Cabernet Sauvignon	2019	14.32±0.00	4.50±0.00	3.71±0.00	6.13±0.06	0.60±0.01	41.04±0.42	50.11±0.30	22.88±0.11
HL-11	Helan	90% Cabernet Sauvignon, 10% Merlot	2018	15.08±0.01	4.33±0.06	4.02±0.00	5.40±0.00	0.71±0.01	47.60±0.07	44.30±0.05	28.35±0.05
YC-1	Xixia	90% Cabernet Sauvignon, 10% Merlot	2018	16.13±0.01	3.73±0.06	3.78±0.00	6.40±0.00	0.64±0.00	42.86±0.32	49.06±0.15	32.94±0.16
YC-2	Xixia	100% Cabernet Sauvignon	2016	14.66±0.01	3.00±0.00	3.80±0.00	5.00±0.00	0.67±0.01	53.68±0.02	38.76±0.04	23.43±0.03
YC-3	Xixia	100% Cabernet Sauvignon	2015	14.10±0.01	2.80±0.00	3.71±0.00	5.10±0.00	0.60±0.00	48.44±0.11	42.64±0.14	27.30±0.12
YC-4	Xixia	100% Cabernet Sauvignon	2016	13.99±0.00	2.60±0.00	3.90±0.00	5.00±0.00	0.66±0.01	58.29±0.10	36.65±0.11	26.11±0.05

NO	Region	Variety	Vintage	Alcohol level (%,*v*/*v*)	Residual sugar (g/L)	pH	Total acidity (g/L)	Volatile acidity (g/L)	*L**	*a**	*b**
YC-5	Xixia	100% Cabernet Sauvignon	2017	14.14±0.01	2.87±0.06	3.99±0.00	4.90±0.00	0.72±0.00	46.60±0.25	44.60±0.10	21.42±0.18
YC-7	Xixia	100% Cabernet Sauvignon	2019	14.70±0.01	2.63±0.06	3.92±0.00	6.00±0.00	0.86±0.01	46.50±0.17	45.49±0.14	26.24±0.12
YC-9	Xixia	100% Cabernet Sauvignon	2018	13.79±0.01	2.47±0.06	3.92±0.00	5.40±0.00	0.75±0.00	52.43±0.09	40.83±0.04	18.61±0.21
YC-10	Xixia	80% Cabernet Sauvignon, 20% Merlot	2015	15.09±0.00	3.77±0.06	3.81±0.01	5.90±0.00	0.79±0.00	45.78±0.13	44.71±0.06	26.79±0.05
YC-12	Xixia	100% Cabernet Sauvignon	2019	15.59±0.01	3.93±0.06	3.54±0.00	7.57±0.06	0.69±0.00	25.82±0.05	61.44±0.07	15.16±0.10
YC-13	Xixia	100% Cabernet Sauvignon	2020	15.42±0.01	3.10±0.1	3.99±0.00	5.50±0.00	0.59±0.01	34.61±0.16	54.11±0.12	14.15±0.08
YC-18	Xixia	100% Cabernet Sauvignon	2020	14.43±0.00	2.90±0.00	4.05±0.01	5.40±0.00	0.74±0.01	46.86±0.16	44.95±0.17	23.54±0.21
YC-19	Xixia	100% Cabernet Sauvignon	2017	14.16±0.01	3.03±0.06	3.84±0.01	5.17±0.06	0.61±0.00	59.86±0.17	34.82±0.10	23.09±0.01
YC-21	Xixia	75% Cabernet Sauvignon, 25% Merlot	2016	14.62±0.01	2.87±0.06	3.80±0.00	5.80±0.00	0.67±0.00	50.95±0.33	39.83±0.29	31.77±0.16
YC-22	Xixia	100% Cabernet Sauvignon	2015	14.52±0.00	2.93±0.06	3.89±0.00	5.40±0.00	0.57±0.01	53.03±0.09	37.85±0.10	31.82±0.08
YC-24	Xixia	Cabernet Sauvignon, Merlot, Cabernet Gernischet	2017	14.71±0.00	3.30±0.00	3.78±0.00	5.60±0.00	0.56±0.00	54.53±0.21	40.28±0.23	23.46±0.09
YC-25	Xixia	Cabernet Sauvignon, Merlot, Cabernet Gernischet	2016	15.47±0.00	3.37±0.06	3.82±0.01	5.57±0.06	0.58±0.01	51.95±0.16	41.47±0.13	27.26±0.12
YC-27	Xixia	100% Cabernet Sauvignon	2018	15.68±0.00	4.13±0.06	3.81±0.00	6.00±0.00	0.70±0.01	34.79±0.20	52.70±0.23	19.00±0.06
YC-28	Xixia	90% Cabernet Sauvignon, 10% Merlot	2017	15.05±0.01	5.13±0.06	3.98±0.01	5.80±0.00	0.75±0.00	45.97±0.19	45.72±0.17	26.65±0.06
YC-29	Xixia	90% Cabernet Sauvignon, 10% Merlot	2018	14.46±0.00	3.53±0.06	3.62±0.00	6.50±0.00	0.66±0.01	56.73±0.15	40.85±0.12	22.87±0.08
YC-30	Xixia	100% Cabernet Sauvignon	2015	15.37±0.01	3.40±0.00	3.88±0.00	6.00±0.00	1.02±0.00	50.64±0.29	42.39±0.22	33.17±0.05
YC-31	Xixia	100% Cabernet Sauvignon	2018	14.81±0.00	2.10±0.00	3.76±0.00	6.13±0.06	0.81±0.01	63.15±0.04	31.92±0.02	26.09±0.05
YC-32	Xixia	100% Cabernet Sauvignon	2019	15.22±0.00	5.23±0.06	3.72±0.01	6.30±0.00	0.76±0.01	23.22±0.11	62.94±0.13	10.59±0.13

NO	Region	Variety	Vintage	Alcohol level (%,*v*/*v*)	Residual sugar (g/L)	pH	Total acidity (g/L)	Volatile acidity (g/L)	*L**	*a**	*b**
YC-33	Xixia	100% Cabernet Sauvignon	2017	14.61±0.01	2.53±0.06	4.01±0.00	5.00±0.00	0.74±0.00	57.81±0.03	36.13±0.04	28.79±0.05
YN-1	Yongning	100% Cabernet Sauvignon	2019	15.38±0.01	3.93±0.06	3.76±0.01	5.60±0.00	0.72±0.00	50.08±0.18	45.14±0.11	23.72±0.08
YN-2	Yongning	100% Cabernet Sauvignon	2018	14.73±0.01	4.00±0.00	3.79±0.00	5.10±0.00	0.69±0.01	41.87±0.20	50.05±0.12	16.21±0.06
YN-3	Yongning	100% Cabernet Sauvignon	2017	15.14±0.00	3.70±0.00	3.73±0.00	5.30±0.00	0.74±0.00	57.28±0.11	39.78±0.13	24.48±0.11
YN-4	Yongning	100% Cabernet Sauvignon	2017	14.95±0.00	3.70±0.1.	3.84±0.00	5.13±0.06	0.76±0.01	47.52±0.19	43.15±0.11	21.46±0.11
YN-5	Yongning	100% Cabernet Sauvignon	2018	14.22±0.01	2.97±0.06	3.73±0.00	5.50±0.00	0.60±0.01	50.55±0.22	44.48±0.05	19.65±0.10
YN-6	Yongning	80% Cabernet Sauvignon, 20% Merlot	2016	14.17±0.00	3.90±0.00	3.62±0.00	5.70±0.00	0.49±0.01	57.60±0.27	36.69±0.14	26.78±0.06
YN-7	Yongning	75% Cabernet Sauvignon, 25% Merlot	2017	14.02±0.00	4.03±0.06	3.68±0.00	5.60±0.00	0.53±0.00	57.60±0.35	38.57±0.17	20.47±0.07
YN-8	Yongning	100% Cabernet Sauvignon	2017	13.81±0.00	2.97±0.06	3.65±0.00	5.10±0.00	0.53±0.00	56.02±0.17	43.65±0.10	19.63±0.16
YN-10	Yongning	100% Cabernet Sauvignon	2017	13.97±0.01	3.20±0.00	3.69±0.00	5.30±0.00	0.69±0.00	55.95±0.14	34.63±0.13	27.10±0.09
YN-11	Yongning	100% Cabernet Sauvignon	2018	13.89±0.01	3.23±0.06	3.69±0.00	5.50±0.00	0.69±0.00	54.04±0.24	34.93±0.18	30.66±0.07
YN-12	Yongning	100% Cabernet Sauvignon	2019	13.14±0.00	3.03±0.06	3.63±0.00	5.30±0.00	0.58±0.00	62.01±0.17	29.36±0.18	26.82±0.20
YN-13	Yongning	100% Cabernet Sauvignon	2016	14.63±0.00	3.23±0.15	3.72±0.00	5.50±0.00	0.64±0.00	50.25±0.15	40.43±0.10	25.67±0.09
YN-15	Yongning	100% Cabernet Sauvignon	2017	15.54±0.01	2.30±0.00	4.10±0.00	5.80±0.00	0.85±0.01	41.05±0.05	46.56±0.03	22.75±0.10
YN-16	Yongning	100% Cabernet Sauvignon	2019	14.10±0.01	3.07±0.06	3.72±0.00	6.00±0.00	0.53±0.01	36.60±0.30	52.06±0.27	12.95±0.23
YN-17	Yongning	100% Cabernet Sauvignon	2020	13.36±0.00	3.10±0.00	3.68±0.00	5.40±0.00	0.59±0.01	47.05±0.23	46.07±0.21	10.87±0.16
YN-18	Yongning	100% Cabernet Sauvignon	2017	13.50±0.01	3.00±0.10	3.60±0.00	5.60±0.00	0.60±0.01	49.62±0.06	40.48±0.05	22.43±0.11
YN-19	Yongning	100% Cabernet Sauvignon	2018	13.35±0.01	1.73±0.06	3.77±0.00	5.20±0.00	0.66±0.01	56.41±0.14	38.55±0.11	19.10±0.16

NO	Region	Variety	Vintage	Alcohol level (%,*v*/*v*)	Residual sugar (g/L)	pH	Total acidity (g/L)	Volatile acidity (g/L)	*L**	*a**	*b**
YN-21	Yongning	80% Cabernet Sauvignon, 20% Merlot	2016	15.56±0.00	3.70±0.00	3.83±0.00	6.00±0.00	0.62±0.00	38.82±0.26	47.81±0.21	28.51±0.05
YN-22	Yongning	100% Cabernet Sauvignon	2017	14.83±0.01	2.10±0.10	4.23±0.00	5.27±0.06	0.78±0.01	47.39±0.15	39.70±0.12	21.28±0.10
YN-23	Yongning	100% Cabernet Sauvignon	2018	14.74±0.01	4.43±0.06	4.12±0.00	5.00±0.00	0.72±0.01	50.39±0.17	38.17±0.12	17.33±0.13
YN-24	Yongning	100% Cabernet Sauvignon	2018	14.96±0.01	2.73±0.06	3.96±0.00	5.57±0.06	0.86±0.01	42.90±0.21	46.60±0.14	23.87±0.09
YN-25	Yongning	100% Cabernet Sauvignon	2019	15.19±0.01	3.77±0.06	3.58±0.00	6.90±0.00	0.72±0.01	25.41±0.18	61.38±0.11	13.56±0.14
QTX-3	Qingtongxia	100% Cabernet Sauvignon	2019	14.77±0.00	3.93±0.05	4.01±0.00	5.30±0.00	0.75±0.00	40.86±0.08	46.73±0.13	21.54±0.07
QTX-4	Qingtongxia	100% Cabernet Sauvignon	2020	13.83±0.01	2.97±0.05	3.85±0.00	5.10±0.00	0.50±0.00	43.34±0.11	46.14±0.09	16.54±0.12
QTX-5	Qingtongxia	90% Cabernet Sauvignon, 10% Merlot	2017	15.40±0.00	1.83±0.06	4.13±0.00	5.70±0.00	0.70±0.01	41.32±2.19	46.89±0.65	25.94±1.58
QTX-6	Qingtongxia	90% Cabernet Sauvignon, 10% Merlot	2018	13.53±0.01	2.20±0.10	3.81±0.01	5.80±0.00	0.60±0.00	64.58±0.22	32.88±0.21	23.04±0.18
QTX-7	Qingtongxia	100% Cabernet Sauvignon	2016	15.13±0.01	3.90±0.00	3.81±0.00	5.60±0.00	0.72±0.01	43.54±0.24	47.02±0.18	22.45±0.02
QTX-9	Qingtongxia	100% Cabernet Sauvignon	2017	14.23±0.00	2.27±0.06	3.98±0.00	5.30±0.00	0.70±0.01	56.23±0.19	36.50±0.18	18.04±0.04
QTX-11	Qingtongxia	Cabernet Sauvignon, Syrah, Merlot	2018	14.99±0.01	2.90±0.00	3.93±0.00	6.20±0.00	0.90±0.01	44.03±0.17	45.79±0.05	24.05±0.03
QTX-13	Qingtongxia	100% Cabernet Sauvignon	2015	13.81±0.00	3.80±0.00	3.76±0.00	5.60±0.00	0.58±0.00	61.66±0.22	33.25±0.14	28.21±0.13
QYX-17	Qingtongxia	100% Cabernet Sauvignon	2018	14.22±0.00	3.20±0.10	3.63±0.00	5.80±0.00	0.59±0.01	54.72±0.16	42.66±0.09	19.63±0.12
HSP-1	Hongsipu	90% Cabernet Sauvignon, 10% Merlot	2018	13.29±0.01	4.90±0.00	3.70±0.00	5.30±0.00	0.56±0.01	59.92±0.11	37.81±0.05	18.26±0.25
HSP-2	Hongsipu	90% Cabernet Sauvignon, 10% Merlot	2019	14.36±0.01	4.50±0.00	3.58±0.01	6.60±0.00	0.57±0.01	32.29±0.26	55.05±0.11	14.75±0.22
HSP-3	Hongsipu	100% Cabernet Sauvignon	2016	14.87±0.01	3.30±0.10	3.76±0.01	5.70±0.00	0.56±0.00	50.19±0.22	42.81±0.18	26.34±0.20

Table S1B. The statistical summary of basic physical and chemical indexes of Cabernet Sauvignon wines from six sub-regions of EFHM.

	Shizuishan	Helan	Xixia	Yongning	Qingtongxia	Hongsipu
Alcohol level (%,*v*/*v*)	14.60±0.14^{ab}	15.18±0.77^{a}	14.82±0.61^{ab}	14.47±0.77^{ab}	14.42±0.67^{ab}	14.20±0.82^{b}
Residual sugar (g/L)	3.45±0.49^{a}	4.25±2.27^{a}	3.27±0.80^{a}	3.28±0.63^{a}	3.01±0.80^{a}	4.20±0.89^{a}
pH	3.9±0.16^{a}	3.87±0.12^{ab}	3.84±0.13^{ab}	3.78±0.17^{ab}	3.88±0.15^{a}	3.68±0.09^{b}
Total acidity (g/L)	5.60±0.14^{a}	5.96±0.60^{a}	5.72±0.63^{a}	5.55±0.43^{a}	5.60±0.33^{a}	5.87±0.67^{a}
Volatile acidity (g/L)	0.85±0.07^{a}	0.65±0.09^{b}	0.71±0.11^{b}	0.67±0.12^{b}	0.68±0.12^{b}	0.60±0.00^{b}

* Averages and standard deviation followed by different letters are significant at $p < 0.05$, Duncan’s multiple range test.

Table S2A. The materials and scales for training in CATA.

sensation options	Description	Training Mode
**Appearance**		
brick red		
brown		
garnet		
light ruby		
ruby		
deep ruby		
light purple		
purple		
deep purple		
**Astringency strength**	Sensation of drying or mouth puckering	
weak		0.1 g/L skin tannin aqueous solutions
weak moderate		0.5 g/L skin tannin aqueous solutions
moderate		1.0 g/L skin tannin aqueous solutions
moderately strong		1.5 g/L skin tannin aqueous solutions
strong		2.0 g/L skin tannin aqueous solutions
**Tannin texture**	Tannin perception in the mouth	
satin		Satin cloth
velvet		Velvet cloth
fine emery		1000 Grade emery paper
abrasive		600 Grade sandpaper

Table S2B. The CATA frequency of 71 Cabernet Sauvignon wines from the six sub-regions of EFHM.

sample	deep purple	purple	light purple	deep ruby	ruby	light ruby	garnet	brown	brick red	satin	velvet	fine emery	abrasive	weak	weak moderate	moderate	moderately strong	strong
SZS-1	0.00	0.03	0.00	0.13	**0.20**	0.13	0.18	0.18	0.18	0.18	**0.53**	**0.25**	0.05	**0.25**	**0.35**	**0.23**	0.15	0.03
SZS-2	0.00	0.10	0.05	0.08	**0.48**	0.13	0.10	0.03	0.05	**0.20**	**0.25**	**0.43**	0.13	0.08	**0.28**	**0.38**	**0.28**	0.00
HL-1	0.00	0.03	0.00	0.18	0.18	0.10	0.15	0.15	**0.23**	0.18	**0.48**	**0.35**	0.00	0.18	**0.30**	**0.38**	0.13	0.03
HL-2*	0.00	0.00	0.03	0.05	**0.35**	**0.23**	0.18	0.13	0.05	0.18	**0.40**	**0.38**	0.05	**0.20**	**0.35**	**0.35**	0.10	0.00
HL-3	0.00	0.10	0.00	**0.20**	**0.33**	0.15	0.13	0.05	0.05	**0.28**	**0.33**	**0.38**	0.03	**0.23**	**0.45**	**0.20**	0.13	0.00
HL-4*	0.10	0.08	0.03	**0.30**	**0.33**	0.15	0.03	0.00	0.00	0.13	**0.30**	**0.48**	0.10	0.10	**0.30**	**0.25**	**0.25**	0.10
HL-5*	**0.38**	**0.23**	0.13	**0.23**	0.05	0.00	0.00	0.00	0.00	0.18	**0.35**	**0.38**	0.10	0.15	**0.20**	**0.20**	**0.35**	0.10
HL-6*	0.10	**0.33**	0.20	0.18	**0.20**	0.00	0.00	0.00	0.00	0.18	**0.35**	**0.45**	0.03	**0.20**	**0.20**	**0.38**	**0.23**	0.00
HL-7	0.15	**0.23**	0.18	0.18	**0.20**	0.03	0.05	0.00	0.00	0.15	**0.40**	**0.45**	0.00	0.13	**0.28**	**0.40**	0.18	0.03
HL-8	0.00	0.00	0.00	0.05	**0.38**	**0.25**	**0.20**	0.05	0.08	0.18	**0.35**	**0.43**	0.05	0.15	**0.23**	**0.30**	**0.25**	0.08
HL-9	**0.28**	0.13	0.00	**0.40**	0.15	0.00	0.05	0.00	0.00	**0.28**	**0.40**	**0.28**	0.05	0.18	**0.33**	**0.30**	0.18	0.03
HL-10*	0.05	0.05	0.03	**0.30**	**0.40**	0.08	0.05	0.03	0.03	0.18	**0.40**	**0.43**	0.00	0.15	**0.33**	**0.38**	0.15	0.00
HL-11	0.00	0.00	0.03	0.10	**0.28**	0.18	**0.28**	0.08	0.08	0.08	0.40	**0.38**	0.15	0.18	**0.28**	**0.25**	**0.20**	0.10
YC-1*	0.00	0.00	0.00	**0.20**	**0.28**	0.15	**0.23**	0.08	0.08	0.05	**0.55**	**0.38**	0.03	**0.20**	**0.40**	**0.33**	0.08	0.00
YC-2*	0.03	0.08	0.10	**0.28**	**0.25**	0.18	0.05	0.05	0.00	**0.23**	**0.25**	**0.50**	0.03	**0.25**	**0.33**	**0.30**	0.13	0.00
YC-3*	0.03	0.08	0.03	**0.33**	**0.28**	0.08	0.15	0.03	0.03	**0.20**	**0.33**	**0.35**	0.13	**0.20**	**0.28**	**0.30**	**0.20**	0.03
YC-4	0.00	0.00	0.05	0.03	**0.23**	**0.23**	**0.28**	0.10	0.10	0.15	**0.40**	**0.40**	0.05	0.15	**0.25**	**0.28**	**0.25**	0.08
YC-5	0.05	0.13	0.10	**0.30**	**0.30**	0.13	0.00	0.00	0.00	0.18	**0.28**	**0.40**	0.15	0.13	**0.23**	**0.40**	**0.23**	0.03
YC-7*	0.03	0.00	0.03	**0.38**	**0.30**	0.18	0.03	0.03	0.05	0.10	**0.38**	**0.35**	0.18	**0.25**	0.18	**0.38**	0.15	0.05
YC-9*	0.00	0.13	**0.28**	0.08	**0.28**	0.18	0.08	0.00	0.00	**0.25**	**0.60**	0.10	0.05	**0.33**	**0.40**	**0.25**	0.03	0.00
YC-12*	**0.25**	**0.30**	0.08	**0.30**	0.08	0.00	0.00	0.00	0.00	0.13	**0.23**	**0.40**	**0.25**	0.05	**0.20**	**0.33**	**0.33**	0.10
YC-13*	**0.20**	**0.30**	0.13	**0.30**	0.03	0.03	0.03	0.00	0.00	0.10	**0.33**	**0.40**	0.18	0.10	0.18	**0.30**	**0.33**	0.10
YC-18	0.08	0.10	0.10	0.13	**0.45**	0.08	0.03	0.00	0.05	**0.28**	**0.30**	**0.38**	0.05	**0.23**	**0.30**	**0.35**	0.10	0.03

sample	deep purple	purple	light purple	deep ruby	ruby	light ruby	garnet	brown	brick red	satin	velvet	fine emery	abrasive	weak	weak moderate	moderate	moderately strong	strong
YC-19	0.00	0.03	0.05	0.05	**0.28**	**0.20**	**0.28**	0.05	0.08	**0.25**	**0.48**	**0.28**	0.00	**0.33**	**0.35**	0.15	0.15	0.03
YC-21	0.03	0.05	0.00	**0.25**	0.15	0.18	0.10	**0.23**	0.03	**0.20**	**0.40**	**0.38**	0.03	0.05	**0.38**	**0.35**	0.18	0.05
YC-22	0.05	0.00	0.03	**0.38**	0.15	0.08	0.15	0.13	0.05	0.10	**0.43**	**0.43**	0.05	0.15	**0.25**	**0.35**	**0.23**	0.03
YC-24	0.00	0.05	0.00	0.08	**0.33**	**0.28**	0.18	0.03	0.08	0.13	**0.38**	**0.43**	0.08	0.10	**0.33**	**0.35**	**0.20**	0.03
YC-25	0.03	0.05	0.03	0.13	**0.35**	0.08	**0.23**	0.13	0.00	0.10	**0.38**	**0.50**	0.03	0.18	**0.28**	**0.35**	0.15	0.05
YC-27	**0.33**	**0.35**	0.10	**0.20**	0.00	0.03	0.00	0.00	0.00	0.13	**0.48**	**0.35**	0.05	0.08	**0.35**	**0.35**	0.15	0.08
YC-28	0.03	0.00	0.03	**0.30**	**0.35**	0.13	0.03	0.08	0.08	**0.25**	**0.28**	**0.48**	0.00	0.15	**0.33**	**0.40**	0.10	0.03
YC-29	0.05	0.00	0.08	0.05	**0.30**	**0.38**	0.08	0.05	0.03	**0.23**	**0.40**	**0.33**	0.05	0.13	**0.48**	**0.30**	0.08	0.03
YC-30*	0.08	0.00	0.03	**0.43**	**0.20**	0.05	0.15	0.03	0.05	0.08	**0.30**	**0.53**	0.10	0.05	0.15	**0.55**	0.15	0.10
YC-31	0.03	0.08	0.08	0.08	**0.28**	**0.30**	0.08	0.08	0.03	0.15	**0.35**	**0.43**	0.08	0.08	**0.25**	**0.48**	0.18	0.03
YC-32*	**0.65**	0.13	0.10	0.10	0.00	0.00	0.03	0.00	0.00	0.03	**0.28**	**0.43**	**0.28**	0.08	**0.23**	**0.20**	**0.38**	0.13
YC-33*	0.00	0.03	0.05	0.10	**0.28**	0.13	0.13	**0.20**	0.10	**0.33**	**0.53**	0.13	0.03	**0.38**	**0.38**	**0.20**	0.03	0.03
YN-1*	0.00	0.00	0.10	0.08	**0.33**	0.18	**0.25**	0.08	0.00	**0.20**	**0.58**	0.18	0.05	**0.30**	**0.38**	0.18	0.15	0.00
YN-2*	**0.03**	0.18	**0.20**	**0.25**	**0.23**	0.08	0.05	0.00	0.00	**0.23**	**0.45**	**0.30**	0.03	**0.33**	**0.43**	**0.23**	0.03	0.00
YN-3	0.00	0.00	0.05	0.03	**0.28**	**0.23**	**0.20**	0.13	0.10	**0.20**	**0.33**	**0.38**	0.10	**0.20**	**0.38**	**0.30**	0.10	0.03
YN-4*	0.03	0.13	0.08	**0.33**	**0.20**	0.05	0.13	0.03	0.05	0.13	**0.30**	**0.40**	0.18	0.18	0.18	**0.33**	**0.30**	0.03
YN-5	0.10	0.08	0.10	0.13	**0.33**	0.15	0.08	0.05	0.00	0.15	**0.55**	**0.28**	0.03	0.18	**0.40**	**0.35**	0.08	0.00
YN-6	0.00	0.05	0.05	0.05	**0.25**	**0.28**	0.18	0.10	0.05	0.08	**0.33**	**0.50**	0.10	**0.23**	**0.25**	**0.38**	0.13	0.03
YN-7	0.00	0.03	0.13	0.03	**0.25**	**0.30**	**0.23**	0.03	0.03	0.18	**0.60**	**0.23**	0.00	**0.35**	**0.30**	**0.33**	0.03	0.00
YN-8*	0.05	0.13	0.13	0.15	**0.25**	0.18	0.03	0.08	0.03	0.15	**0.33**	**0.45**	0.08	**0.25**	**0.30**	**0.30**	0.13	0.03
YN-10	0.03	0.03	0.03	**0.25**	**0.20**	0.05	**0.25**	0.08	0.10	0.18	**0.50**	**0.23**	0.10	**0.40**	**0.20**	**0.28**	0.10	0.03
YN-12	0.00	0.00	0.00	0.03	0.03	**0.23**	**0.25**	**0.35**	0.13	**0.20**	**0.35**	**0.43**	0.03	0.18	**0.28**	**0.35**	**0.20**	0.00
YN-13	0.05	0.03	0.05	**0.28**	0.18	**0.25**	0.18	0.00	0.00	0.13	**0.30**	**0.53**	0.05	**0.25**	0.15	**0.33**	**0.25**	0.03
YN-15	0.15	0.05	0.00	**0.40**	**0.23**	0.10	0.05	0.03	0.00	**0.20**	**0.43**	**0.35**	0.03	0.18	**0.20**	**0.45**	0.13	0.05

sample	deep purple	purple	light purple	deep ruby	ruby	light ruby	garnet	brown	brick red	satin	velvet	fine emery	abrasive	weak	weak moderate	moderate	moderately strong	strong
YN-16	0.18	**0.25**	0.20	**0.23**	0.15	0.00	0.00	0.00	0.00	0.08	**0.43**	**0.43**	0.08	**0.25**	**0.23**	**0.33**	0.18	0.03
YN-17	0.03	**0.33**	**0.30**	0.10	0.13	0.03	0.10	0.00	0.00	**0.30**	**0.33**	**0.25**	0.13	**0.25**	**0.28**	**0.30**	0.18	0.00
YN-18	0.05	0.05	0.03	**0.43**	0.18	0.10	0.10	0.03	0.05	**0.20**	**0.35**	**0.40**	0.05	**0.20**	**0.25**	**0.38**	0.18	0.00
YN-19	0.00	0.00	0.08	0.08	**0.38**	0.18	**0.25**	0.05	0.00	0.18	**0.50**	**0.23**	0.10	0.18	**0.40**	**0.25**	0.18	0.00
YN-20	0.08	0.08	0.00	**0.45**	0.13	0.03	0.03	0.13	0.10	**0.20**	0.18	**0.40**	**0.23**	0.08	0.10	**0.33**	**0.33**	0.18
YN-21	0.10	0.05	0.00	**0.38**	0.18	0.03	0.13	0.10	0.05	**0.23**	**0.28**	**0.43**	0.08	0.08	**0.35**	**0.45**	0.10	0.03
YN-22	0.05	0.00	0.03	**0.20**	**0.35**	0.10	0.13	0.08	0.08	**0.28**	**0.43**	**0.30**	0.00	0.10	**0.30**	**0.43**	0.18	0.00
YN-23*	0.05	0.10	0.10	0.15	**0.28**	**0.20**	0.08	0.03	0.03	**0.28**	**0.50**	0.15	0.08	**0.23**	**0.35**	**0.35**	0.08	0.00
YN-24	0.08	0.18	0.05	**0.33**	0.18	0.10	0.08	0.00	0.03	0.08	**0.48**	**0.38**	0.08	0.13	**0.40**	**0.33**	0.15	0.00
YN-25*	**0.35**	**0.33**	0.10	0.10	0.05	0.00	0.08	0.00	0.00	0.10	**0.33**	**0.48**	0.10	0.10	0.08	**0.38**	**0.33**	0.13
QTX-3	0.18	**0.30**	0.15	**0.25**	0.08	0.03	0.03	0.00	0.00	**0.28**	**0.35**	**0.33**	0.05	0.15	**0.35**	**0.30**	0.15	0.05
QTX-4	0.13	**0.40**	**0.25**	0.10	0.08	0.03	0.03	0.00	0.00	**0.20**	**0.28**	**0.43**	0.10	**0.20**	**0.20**	**0.20**	**0.35**	0.05
QTX-5*	0.10	0.10	0.08	**0.35**	**0.23**	0.05	0.08	0.00	0.03	**0.43**	**0.30**	**0.25**	0.03	**0.25**	**0.33**	**0.33**	0.10	0.00
QTX-6	0.00	0.00	0.03	0.00	0.05	**0.28**	**0.25**	**0.38**	0.03	**0.40**	**0.33**	**0.23**	0.05	**0.25**	**0.48**	0.18	0.10	0.00
QTX-7*	0.03	0.08	0.13	**0.28**	0.38	0.03	0.05	0.03	0.03	**0.28**	**0.45**	**0.25**	0.03	**0.20**	**0.25**	**0.43**	0.13	0.00
QTX-9*	0.05	0.13	0.15	**0.23**	**0.23**	0.10	0.05	0.03	0.05	**0.28**	**0.40**	**0.28**	0.05	**0.25**	**0.30**	**0.35**	0.10	0.00
QTX-11	**0.20**	**0.20**	0.10	**0.30**	0.08	0.03	0.05	0.00	0.05	0.18	**0.40**	**0.35**	0.08	**0.20**	**0.35**	**0.28**	0.13	0.05
QTX-13*	0.00	0.00	0.00	0.00	**0.28**	**0.25**	0.10	**0.23**	0.15	**0.40**	**0.43**	0.18	0.00	**0.30**	**0.38**	**0.20**	0.13	0.00
QTX-17	0.00	0.05	0.05	0.03	**0.40**	**0.20**	**0.23**	0.03	0.03	**0.28**	**0.40**	**0.28**	0.05	**0.20**	**0.45**	**0.23**	0.13	0.00
HSP-1*	0.00	0.00	0.13	0.03	**0.33**	**0.23**	0.13	0.13	0.05	**0.25**	**0.25**	**0.40**	0.10	**0.23**	**0.30**	**0.40**	0.08	0.00
HSP-2*	**0.25**	**0.53**	0.03	0.10	0.10	0.00	0.00	0.00	0.00	0.10	0.15	**0.60**	0.15	0.10	0.13	**0.38**	**0.33**	0.08
HSP-3*	0.03	0.08	0.08	**0.20**	**0.43**	0.10	0.05	0.00	0.05	**0.25**	**0.20**	**0.48**	0.08	0.10	**0.25**	**0.45**	0.18	0.03

*The representative wines of each sub-region were selected and evaluated for QDA.

Table S2C. The QDA result of 71 Cabernet Sauvignon wines from the six sub-regions of EFHM.

	Helan	Xixia	Yongning	Qingtongxia	Hongsipu
Astringency strength	3.6±1.2^{b}	4.2±1.3^{ab}	4.2±1.2^{ab}	4.6±1.4^{a}	4.8±1.4^{a}
Tannin texture	5.6±0.9^{a}	5.3±1.0^{ab}	5.6±1.0^{a}	5.5±0.9^{a}	5.1±0.9^{b}
Body	5.6±1.0^{a}	5.6±1.1^{a}	5.5±1.1^{a}	5.6±0.9^{a}	5.6±0.9^{a}
Finish	5.4±1.0^{b}	5.6±1.2^{ab}	5.6±1.1^{ab}	5.7±1.1^{ab}	5.8±1.2^{a}

* Averages and standard deviation followed by different letters are significant different at significant level of p < 0.05 using Duncan's multiple range test.

Table S3. The historic terroir parameter of the six sub-regions of EFHM.

Sub-regions	Altitude (m)	Latitude and longitude	Duration of sunshine (h)	Effective accumulated temperature (°C)			Rainfall (mm)		
			From April to October	From April to October	From July to October	Annual	From April to October	From July to October	Annual
Shizuishan	1117.7	39°03' N 106°35' E	1600.28	1856.28	1086.41	1913.37	170.88	120.91	194.91
Helan	1106.2	38°57' N 106°35' E	1681.69	1699.65	1007.47	1746.70	170.97	114.29	199.67
Xixia	1110.9	38°47' N 106°20' E	1569.81	1666.21	996.99	1716.40	187.70	123.60	215.06
Yongning	1113.7	38°28' N 106°25' E	1604.38	1666.74	989.45	1720.55	170.53	107.68	197.92
Qingtongxia	1118.0	38°03' N 106°08' E	1688.00	1658.97	975.95	1711.87	181.66	122.15	207.35
Hongsipu	1128.8	37°98' N 106°18' E	1676.69	1715.36	1013.00	1774.26	183.73	122.53	212.24

a. The data is from 1982 to 2011 in local meteorological.

b. The grape growing season in the eastern foothill of Helan Mountain is from April to October.

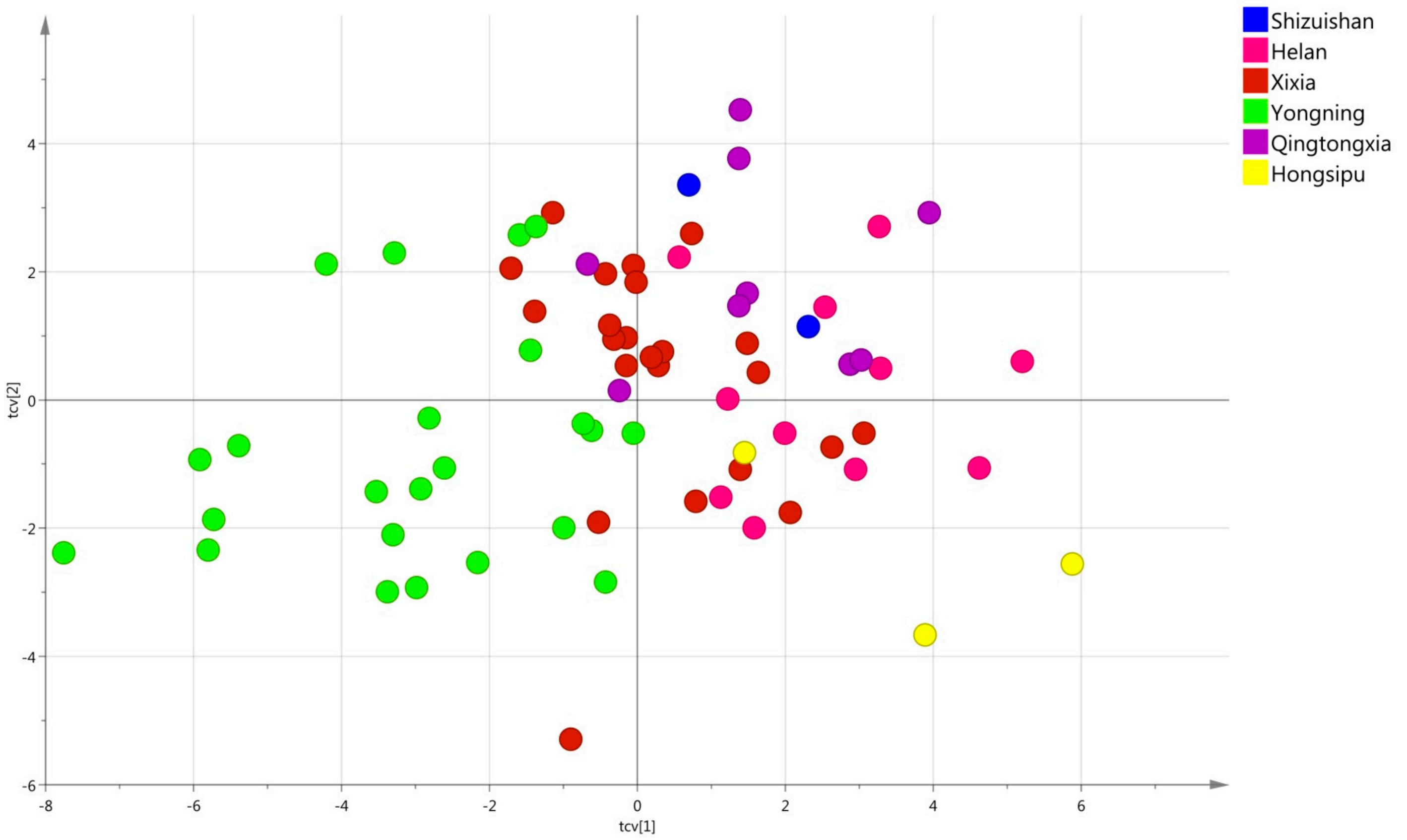

Figure S1. Cross-validated score plot for the OPLS-DA model based on the concentrations of phenolic compounds in Cabernet Sauvignon wines from six sub-regions of EFHM.

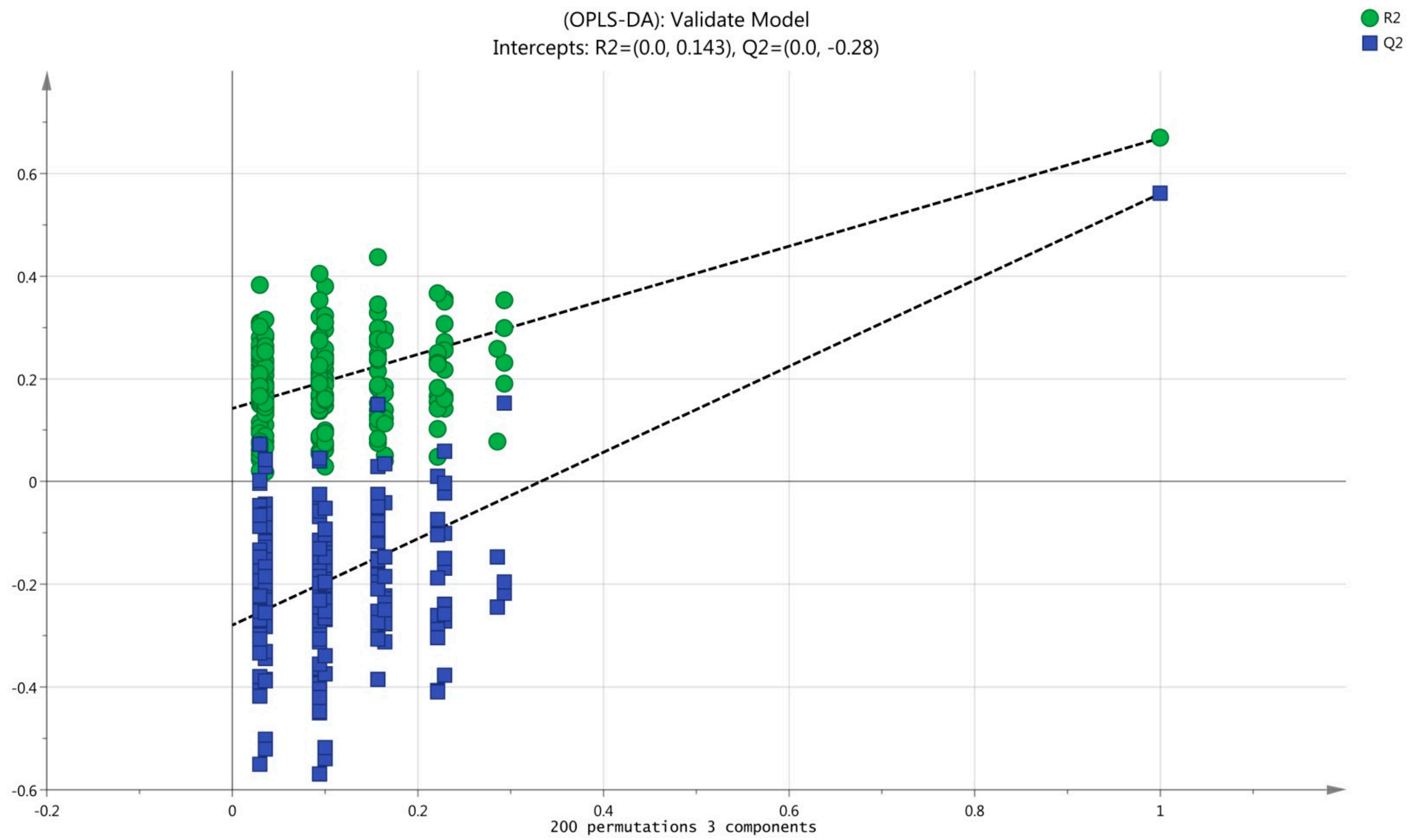

Figure S2. Validation plot obtained from 200-time permutation tests for the OPLS-DA model based on the concentrations of phenolic compounds in Cabernet Sauvignon wines from six sub-regions of EFHM.

Table S4. Concentration of non-anthocyanin phenolic compound in Cabernet Sauvignon wines from six sub-regions of EFHM.

Non-anthocyanin phenolic compounds (mg/L)	Shizuishan	Helan	Xixia	Yongning	Qingtongxia	Hongsipu
**Flavan-3-ols**	**375.01±127.31[a]**	**376.43±83.29[a]**	**382.47±153.02[a]**	**315.14±109.58[a]**	**400.47±125.14[a]**	**449.13±154.40[a]**
PC B	238.4±100.58[a]	244.32±58.53[a]	247.36±100.98[a]	203.78±77.12[a]	260.80±92.19[a]	301.25±112.65[a]
PC C	7.56±6.56[a]	8.95±3.09[a]	8.58±5.08[a]	6.25±3.44[a]	8.69±4.09[a]	11.38±5.23[a]
EGC	7.52±0.83[a]	8.68±2.15[a]	7.52±4.44[a]	6.39±2.66[a]	9.27±2.72[a]	7.86±2.34[a]
C	46.14±11.57[a]	46.60±12.10[a]	49.34±21.22[a]	38.80±11.47[a]	46.02±12.45[a]	48.60±15.57[a]
EC	58.88±9.06[a]	49.80±9.63[a]	52.97±22.42[a]	42.93±12.96[a]	54.26±14.40[a]	61.39±16.24[a]
GC	16.49±0.36[a]	18.08±3.44[a]	16.69±6.86[a]	16.98±7.79[a]	21.42±5.14[a]	18.64±3.64[a]
**Flavonols**	**50.78±20.95[b]**	**78.74±21.84[a]**	**59.78±16.25[ab]**	**56.51±17.54[ab]**	**53.74±8.25[b]**	**49.36±23.92[b]**
M-glu	10.25±14.03[b]	28.99±10.17[a]	18.24±8.71[ab]	18.85±9.45[ab]	9.89±5.31[b]	8.48±9.82[b]
DHQ	1.06±0.07[ab]	1.03±0.11[ab]	0.98±0.21[ab]	0.95±0.17[b]	1.09±0.14[ab]	1.22±0.42[a]
DHK	0.88±0.03[ab]	0.95±0.09[ab]	0.86±0.14[b]	0.84±0.12[b]	0.94±0.09[ab]	1.04±0.29[a]
Q-glu	0.99±1.00[b]	6.82±6.48[a]	1.69±2.60[b]	2.63±3.19[ab]	0.60±0.80[b]	0.40±0.16[b]
Q-gal	1.55±1.77[a]	2.00±1.08[a]	1.55±0.56[a]	1.52±0.5[a]	1.43±0.44[a]	1.32±1.02[a]
Q-gluc	10.97±10.31[a]	12.32±2.52[a]	11.62±3.53[a]	10.94±2.65[a]	11.43±2.38[a]	12.44±4.20[a]
Q	5.84±0.34[a]	9.41±2.87[a]	7.87±3.63[a]	5.67±4.65[a]	9.5±4.12[a]	10.17±7.80[a]
L	1.54±1.28[a]	0.68±0.13[c]	0.79±0.26[bc]	0.68±0.21[c]	1.09±0.37[b]	0.80±0.21[bc]
M	10.58±4.93[a]	7.19±1.95[a]	8.65±3.48[a]	6.83±2.97[a]	10.80±2.58[a]	8.56±5.77[a]
I-glu	1.07±1.11[b]	2.35±1.34[a]	1.18±0.80[b]	1.41±0.82[ab]	0.66±0.50[b]	0.60±0.40[b]
K-glu	0.44±0.02[ab]	0.88±0.77[a]	0.46±0.15[ab]	0.50±0.23[ab]	0.41±0.03[b]	0.40±0.04[b]
S-glu	5.29±1.33[a]	5.40±1.13[a]	5.21±0.97[a]	5.06±0.92[a]	5.27±1.06[a]	3.32±0.88[b]
Q-rha	0.74±0.02[a]	0.72±0.16[a]	0.65±0.08[a]	0.63±0.11[a]	0.63±0.09[a]	0.61±0.10[a]
**Hydroxybenzoic acids**	**38.99±9.58[a]**	**26.74±7.53[b]**	**26.62±7.38[b]**	**33.26±9.24[ab]**	**24.12±9.18[b]**	**38.02±5.66[a]**
CA	tr	tr	tr	tr	tr	tr

Non-anthocyanin phenolic compounds (mg/L)	Shizuishan	Helan	Xixia	Yongning	Qingtongxia	Hongsipu
GLA	29.52±10.63[ab]	22.71±7.01[b]	22.22±6.53[b]	27.06±7.90[ab]	20.16±8.79[b]	33.78±5.13[a]
PA	6.68±0.25[a]	2.12±0.98[b]	2.60±1.57[b]	4.13±3.15[ab]	1.87±1.50[b]	2.86±0.82[b]
4-HBA	1.49±0.52[a]	0.68±0.17[b]	0.76±0.26[b]	0.97±0.42[b]	0.75±0.16[b]	0.63±0.13[b]
GTA	1.09±0.71[a]	1.08±0.43[a]	0.86±0.39[a]	0.92±0.48[a]	1.20±0.60[a]	0.62±0.13[a]
VA	0.22±0.06[a]	0.15±0.02[b]	0.17±0.04[ab]	0.18±0.05[ab]	0.16±0.03[b]	0.14±0.01[b]
**Hydroxycinnamic acids**	**12.87±0.55[a]**	**12.39±5.27[a]**	**11.18±4.85[a]**	**12.22±4.64[a]**	**13.04±3.83[a]**	**6.98±0.42[a]**
CFA	11.70±0.13[a]	11.22±5.09[a]	10.10±4.65[a]	11.15±4.47[a]	11.83±3.57[a]	6.07±0.38[a]
3-HCA	0.55±0.37[a]	0.49±0.12[ab]	0.44±0.15[ab]	0.42±0.10[ab]	0.52±0.20[a]	0.32±0.11[b]
FA	0.62±0.04[a]	0.69±0.17[a]	0.64±0.14[a]	0.64±0.14[a]	0.68±0.19[a]	0.59±0.13[a]

a. Expressed as average value plus and minus standard deviation; different letters in the same row indicated significant difference ($p < 0.05$) using Duncan's multiple range test.

b. Quantitative results that were less than 1.5 times the intercept of the calibration curve were labeled 'trace'.

Table S5. Concentration of anthocyanin in Cabernet Sauvignon wines from six sub-regions of EFHM.

Anthocyanins (mg/L)	Percentage	Shizuishan	Helan	Xixia	Yongning	Qingtongxia	Hongsipu
**Total anthocyanins**		**26.16±9.05[a]**	**50.63±22.22[a]**	**35.83±25.61[a]**	**29.70±26.52[a]**	**44.32±17.78[a]**	**36.81±15.35[a]**
**Total cyanidin**	**(1.3%-2.9%)**	0.43±0.16[b]	0.68±0.30[b]	0.47±0.32[b]	0.37±0.29[b]	0.55±0.27[b]	1.12±0.71[a]
Cy-glu		0.23±0.11[b]	0.38±0.19[b]	0.25±0.18[b]	0.19±0.16[b]	0.28±0.15[b]	0.68±0.46[a]
Cy-Aglu		0.15±0.04[ab]	0.22±0.09[ab]	0.16±0.12[ab]	0.13±0.1[b]	0.21±0.12[ab]	0.29±0.16[a]
Cy-Cglu		0.05±0.01[b]	0.08±0.03[b]	0.06±0.03[b]	0.06±0.03[b]	0.07±0.02[b]	0.15±0.09[a]
**Total delphinidin**	**(6.3%-12.6%)**	**2.08±0.29[b]**	**4.00±2.10[ab]**	**2.60±2.07[ab]**	**1.92±1.85[b]**	**3.35±1.75[ab]**	**4.93±2.77[a]**
Dp-glu		1.61±0.19[b]	3.12±1.71[ab]	1.99±1.61[ab]	1.45±1.44[b]	2.53±1.37[ab]	3.92±2.24[a]
Dp-Aglu		0.41±0.09[a]	0.76±0.35[a]	0.52±0.42[a]	0.39±0.37[a]	0.71±0.39[a]	0.76±0.38[a]
Dp-Cglu		0.06±0.01[b]	0.12±0.06[b]	0.09±0.05[b]	0.08±0.05[b]	0.11±0.04[b]	0.25±0.15[a]
**Total peonidin**	**(7.3%-10.9%)**	**2.28±1.21[a]**	**3.94±1.67[a]**	**2.93±2.36[a]**	**2.24±2.03[a]**	**3.42±1.63[a]**	**4.22±2.34[a]**
Pn-glu		1.41±0.79[a]	2.47±1.09[a]	1.83±1.48[a]	1.37±1.21[a]	2.02±1.01[a]	2.85±1.64[a]
Pn-Aglu		0.68±0.31[a]	1.06±0.41[a]	0.80±0.66[a]	0.63±0.59[a]	1.03±0.57[a]	0.96±0.50[a]
Pn-Cglu		0.19±0.11[a]	0.41±0.20[a]	0.30±0.24[a]	0.25±0.24[a]	0.37±0.17[a]	0.41±0.21[a]
**Total petunidin**	**(7.3%-10.7%)**	**2.00±0.35[a]**	**4.05±1.95[a]**	**2.82±2.19[a]**	**2.22±2.15[a]**	**3.51±1.72[a]**	**4.14±2.20[a]**
Pt-glu		1.35±0.20[a]	2.74±1.33[a]	1.90±1.47[a]	1.48±1.45[a]	2.33±1.18[a]	2.90±1.56[a]
Pt-Aglu		0.56±0.13[a]	1.13±0.54[a]	0.79±0.64[a]	0.62±0.62[a]	1.03±0.55[a]	1.00±0.51[a]
Pt-Cglu		0.08±0.02[b]	0.18±0.10[ab]	0.13±0.09[ab]	0.12±0.10[ab]	0.15±0.06[ab]	0.23±0.13[a]
**Total malvidin**	**(62.9%-76.1%)**	**19.37±7.04[a]**	**37.96±17.06[a]**	**27.01±18.99[a]**	**22.95±20.56[a]**	**33.48±12.86[a]**	**22.39±8.17[a]**
Mv-glu		13.17±4.31[a]	24.86±10.45[a]	18.16±12.38[a]	15.57±13.59[a]	21.91±7.81[a]	15.43±5.27[a]
Mv-Aglu		5.39±2.34[a]	10.76±5.31[a]	7.30±5.47[a]	6.12±5.68[a]	9.64±4.68[a]	5.54±2.32[a]
Mv-Cglu		0.81±0.38[a]	2.34±1.41[a]	1.54±1.27[a]	1.26±1.36[a]	1.93±0.81[a]	1.43±0.61[a]

a. Expressed as average value plus and minus standard deviation; different letters in the same row indicated significant difference ($p < 0.05$) using Duncan's multiple range test.

Table S6. Concentration of anthocyanin derivative in Cabernet Sauvignon wines from six sub-regions of EFHM.

Anthocyanin derivative (mg/L)	Type	Shizuishan	Helan	Xixia	Yongning	Qingtongxia	Hongsipu
**Total anthocyanin derivatives**		**3.84±0.52[a]**	**5.03±1.32[a]**	**4.48±1.29[a]**	**4.55±0.89[a]**	**4.15±0.82[a]**	**5.31±1.36[a]**
(E)cat-Cy	F-A	tr	tr	tr	tr	tr	tr
(E)cat-Mv	F-A	0.38±0.10[b]	0.64±0.17[a]	0.48±0.19[ab]	0.43±0.22[ab]	0.61±0.15[ab]	0.54±0.02[ab]
(E)cat-Pt	F-A	0.07±0.01[b]	0.09±0.02[ab]	0.07±0.02[b]	0.07±0.02[b]	0.08±0.02[b]	0.11±0.03[a]
Mv-(e)cat	A-F	0.20±0.00[ab]	0.23±0.05[a]	0.18±0.05[ab]	0.15±0.05[b]	0.24±0.06[a]	0.20±0.01[ab]
Pn-(e)cat	A-F	tr	tr	tr	tr	tr	tr
Dp-(e)cat	A-F	tr	tr	tr	tr	tr	tr
Pt-(e)cat	A-F	0.05±0.00[bc]	0.05±0.01[bc]	0.05±0.01[bc]	0.05±0.01[c]	0.06±0.01[b]	0.07±0.01[a]
Cy-(e)cat	A-F	tr	tr	tr	tr	tr	tr
Cy-ace	Vitisin	0.08±0.01[bc]	0.08±0.01[bc]	0.09±0.01[bc]	0.11±0.02[a]	0.08±0.01[c]	0.10±0.02[ab]
Dp-ace	Vitisin	0.05±0.00[b]	0.05±0.01[b]	0.06±0.01[b]	0.07±0.01[a]	0.05±0.00[b]	0.05±0.00[b]
Vitisin B	Vitisin	0.27±0.10[a]	0.60±0.50[a]	0.38±0.35[a]	0.35±0.33[a]	0.36±0.32[a]	0.43±0.42[a]
Pt-ace	Vitisin	0.10±0.08[b]	0.21±0.06[a]	0.17±0.06[ab]	0.23±0.10[a]	0.10±0.03[b]	0.16±0.10[ab]
Pn-ace	Vitisin	0.06±0.02[a]	0.10±0.08[a]	0.07±0.04[a]	0.06±0.03[a]	0.06±0.03[a]	0.11±0.11[a]
Cy-py	Vitisin	tr	tr	tr	tr	tr	tr
Dp-py	Vitisin	0.10±0.02[b]	0.14±0.06[b]	0.13±0.06[b]	0.12±0.04[b]	0.09±0.02[b]	0.24±0.10[a]
Vitisin A	Vitisin	0.98±0.34[b]	1.45±0.71[ab]	1.39±0.69[ab]	1.37±0.45[ab]	0.84±0.38[b]	1.81±0.92[a]
Pt-py	Vitisin	0.12±0.02[b]	0.15±0.05[b]	0.15±0.07[b]	0.14±0.04[b]	0.10±0.03[b]	0.28±0.10[a]
Pn-py	Vitisin	0.36±0.03[b]	0.31±0.09[b]	0.35±0.06[b]	0.37±0.06[ab]	0.29±0.04[b]	0.44±0.11[a]
Mv-v-Cat	Flavanyl-pyranoanthocyanin	0.13±0.01[a]	0.19±0.07[a]	0.16±0.07[a]	0.16±0.06[a]	0.10±0.03[a]	0.14±0.04[a]
Pn-v-Cat	Flavanyl-pyranoanthocyanin	tr	tr	tr	tr	tr	tr
Pt-v-Cat	Flavanyl-pyranoanthocyanin	0.06±0.00[a]	0.08±0.02[a]	0.07±0.02[a]	0.06±0.01[a]	0.06±0.01[a]	0.08±0.03[a]
Mv-vcol	Pinotin	0.28±0.16[a]	0.22±0.19[a]	0.21±0.14[a]	0.23±0.24[a]	0.38±0.28[a]	0.10±0.04[a]
Mv-vpol	Pinotin	0.47±0.01[a]	0.38±0.24[a]	0.41±0.23[a]	0.52±0.25[a]	0.58±0.19[a]	0.39±0.14[a]

Anthocyanin derivatives (mg/L)	Type	Shizuishan	Helan	Xixia	Yongning	Qingtongxia	Hongsipu
Mv-vgol	Pinotin	0.07±0.01[a]	0.06±0.01[a]	0.06±0.01[a]	0.06±0.01[a]	0.07±0.01[a]	0.06±0.01[a]

a. Expressed as average value plus and minus standard deviation; different letters in the same row indicated significant difference ($p < 0.05$) using Duncan's multiple range test.

b. Quantitative results that were less than 1.5 times the intercept of the calibration curve were labeled 'trace'.
